# Incidence and outcome of first syncope in primary care: A retrospective cohort study

**DOI:** 10.1186/1471-2296-12-102

**Published:** 2011-09-27

**Authors:** Peter Vanbrabant, Jean Bernard Gillet, Frank Buntinx, Stefaan Bartholomeeusen, Bert Aertgeerts

**Affiliations:** 1 Emergency Department, University hospitals Leuven, Herestraat 49, B-3000 Leuven, Belgium; 2 Department of General Practice and Intego registry, K.U.Leuven, Kapucijnenvoer 33, B-3000 Leuven, Belgium; 3 Department of General Practice, Maastricht University, 6200 MD Maastricht, The Netherlands

**Keywords:** Syncope, risk assessment, primary health care

## Abstract

**Background:**

Assessment of risk for serious cardiovascular outcome after syncope is difficult.

**Objectives:**

To determine the incidence of first syncope in primary care. To investigate the relation between syncope and serious cardiovascular (CV) outcome and serious injury.

**Methods:**

Retrospective cohort study using data from the Intego general practice-based registration network, collecting data from 55 general practices (90 GP's). All patients with a first syncope from 1994 to 2008 were included; five participants without syncope were matched for age and gender for every patient with syncope. The main outcome measures were incidence of first syncope by age and gender and one year risk of serious CV outcome or injury after syncope.

**Results:**

2785 patients with syncope and 13909 matched patients without syncope were included. The overall incidence of a first syncope was 1.91 per 1000 person-years (95% CI 1.83-1.98). The incidence was higher in females (2.42 (95% CI 2.32-2.55) per 1000 person-years) compared to males (1.4 (95% CI 1.32-1.49) per 1000 person-years) and follows a biphasic pattern according to age: a first peak at the age of 15-24 years is followed by a sharp rise above the age of 45. One year serious outcome after syncope was recorded in 12.3% of patients. Increasing age (HR 1.04 (1.03-1.04)), CV comorbidity (HR 3.48 (95% CI 2.48-4.90) and CV risk factors (HR 1.65 (95% CI 1.24-2.18) are associated with serious outcome. Cox regression, adjusting for age, gender, CV comorbidity and risk factors, showed that syncope was an independent risk factor for serious CV outcome or injury (HR 3.99 (95% CI 3.44-4.63)). The other independent risk factors were CV comorbidity (HR 1.81 (95% CI 1.51-2.17)) and age (HR 1.03 (95% CI 1.03-1.04)).

**Conclusions:**

Incidence rate of first syncope in primary care was 1.91 per 1000 person-years. One year risk of serious outcome after syncope was 12.3%. Increasing age, CV comorbidity and risk factors are associated with serious outcome. Compared to a control group, syncope on itself is an independent risk factor for serious outcome (adjusted for age, gender, CV comorbidity and risk factors).

## Background

Syncope is a transient loss of consciousness due to global cerebral hypo-perfusion characterized by rapid onset, short duration and spontaneous complete recovery [1]. Such an event may have multiple possible causes, from benign conditions to life-threatening disease [2]. Most studies on syncope were conducted in emergency departments and general hospitals, but little is known about incidences and outcome in a general population or general practice.

Epidemiological data are scarce and reported as lifetime prevalence and incidence rates. In a general population, lifetime prevalence in a Framingham cohort (3.0-3.5%) was low compared to data from other populations (19-39%) [3-7]. Incidence rates vary from 6.2 to 39.7 per 1000 person-years [8,9]. Recently, these general population incidence rates were analyzed in relation to general practice and emergency department data. Incidences were respectively 9.3 and 0.7 visits per 1000 person-years [9]. In other reports, the percentage of persons experiencing syncope that seek medical attention (seeing a doctor or visiting a hospital) was 37% and 56% [5,8].

Outcome after syncope is related to the cause of syncope. Patients with syncope due to a cardiac cause have higher mortality compared with patients with non-cardiac causes [8]. However, Kapoor showed that syncope in itself is not a risk factor for mortality, but underlying heart disease is [10]. Serious short-term outcome (including death, myocardial infarction, arrhythmia and major therapeutic procedures) after Emergency Department (ED) visit for syncope ranged from 6.1 to 11.5% [11,12]. Long term-outcome (one or two year mortality or severe outcome) ranged from 8.5 to 11.5% [12-14].

Several risk stratification tools to detect patients with syncope at high risk for serious outcome have been developed. Only two clinical decision rules are sufficiently developed for use in practice (level 2 evidence for clinical decision rules): San Francisco Syncope Rule (SFSR) and The Osservatorio Epidemiologico sulla Sincope nel Lazio(OESIL) risk score [15]. However, these two rules showed considerable inconsistency across studies. Recently, the National Institute for Health and Clinical Excellence (NICE) developed, after careful consideration of the evidence, a guideline about the assessment, diagnosis and specialist referral of adults and young people who experienced a transient loss of consciousness [16]. 'Red flags' were determined to identify high risk patients for serious adverse events that should have specialist assessment urgently.

The aim of the study is to determine the incidence of first syncope as presented to GPs and to investigate whether syncope is associated with increased cardiovascular (CV) outcomes and serious injury within a general practice population.

## Methods

In this retrospective cohort study, we examined data from general practices in Belgium providing data to the Intego general practice registration network, from January 1th, 1994 to December 31th, 2008.

### Data source

The Intego general practice registration network is coordinated by the department of general practice of the Katholieke Universiteit Leuven. This database contains anonymised coded diagnoses (both detaiIed and International Classification of Primary Care codes (ICPC-2)) [17], laboratory results and drug prescriptions. Several studies are already published based on this database [18-20]. Its validity was proven in a recent article [21]. A total of 55 practices (90 GPs) located throughout Flanders collaborate in this data collection process. GPs present themselves for inclusion in the registry, but before their data are accepted, their registration performance is audited using a number of algorithms that compares their results with all other applicants. Only the data of the practices with the best performance (from less than 50% of the applicants) are included in the database. Incidence rates calculation requires knowing the size of the population (denominator). Since people are only partially registered with a particular GP in Belgium and may consult several different doctors, the practice population is unknown. However, the yearly contact group (the group of patients that consulted their GP at least once a year) can easily be obtained from the Intego database. Moreover, a reliable estimate of the Intego practice population (corresponding to 1.72% of the population of Flanders) can be obtained by extrapolating the yearly contact group by a correction factor based on social security data [22].

### Participants

'Exposed' participants: We included all patients with a first syncope, fainting, blackout, vasovagal reaction or collapse (A06 ICPC-2 code) during the period January 1994 to December 2008. Date of diagnosis (A06 ICPC-2 code) was used as the baseline date.

'Unexposed' participants: For each exposed patient 5 (randomly selected) additional patients without syncope were matched with respect to age and gender. They received a baseline date which was similar to the day of diagnosis of the related exposed patient.

### Variables

The (serious) outcome was defined as the occurrence of a new CV event or serious injury within one year. New CV events were myocardial infarction, arrhythmia, pulmonary embolism, stroke, subarachnoid haemorrhage [11]. Serious injury was defined as fractures and intracranial hemorrhage.

In both groups CV comorbidity prior to the event (myocardial infarction, angina, stroke or TIA and congestive heart failure) and known CV risk factors (hypertension, diabetes mellitus and hypercholesterolemia) were recorded.

### Statistical analysis

We calculated incidences (and 95% confidence intervals(CI)) as the number of first syncope-registrations per 1000 patient-years in the practice population.

Survival analyses were performed to take account of the censored nature of the data, whereby the date of diagnosis of the exposed participants is used as the date of origin. For the unexposed participants, the diagnosis date of the matching exposed participant was used. For censored observations (no serious outcome within one year) only the last year of contact could be obtained, not the exact date. Hence, for those patients, the last date of contact was set to 31 December of that year.

Kaplan- Meier curves were calculated for serious outcome for patients with and without syncope. Logrank test was used for comparison of survival curves.

In addition, we used Cox regression analysis to examine differences in outcome, adjusted for age, gender, CV comorbidity and risk factors, and taking possible interactions into consideration.

All analyses have been performed using MedCalc for windows version, 11.3.0. (MedCalc Software, Mariakerke, Belgium) and SPSS for windows version 18.0 (SPSS, Chicago, IL, USA).

## Results

### Demographics

On average 185 patients with first syncope were recorded yearly over the period 1994-2008. This signifies 2785 patients with syncope and 13909 control patients. Follow up was incomplete (less than one year follow-up without reaching the outcome measure) in 394 of the syncope patients (14.7%) and 4352 of the control patients (31.2%).

### Incidence

**Figure 1:**
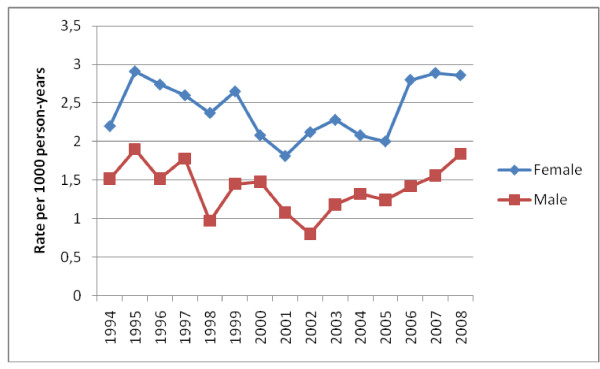
**Annual incidence of first syncope, from 1994 to 2008**.

**Figure 2:**
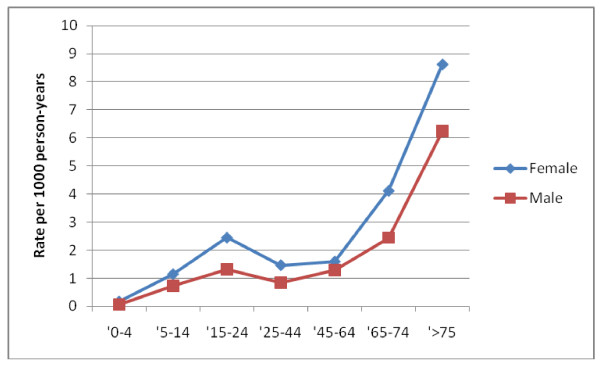
**Incidence of first syncope according to age group**.

The overall incidence of a first syncope was 1.91 per 1000 person-years (95% CI 1.83-1.98). The incidence in females was higher (2.42 (95% CI 2.32-2.55) per 1000 person-years), as compared to males (1.4 (95% CI 1.32-1.49) per 1000 person years). The annual incidence rates over the study period vary from 0.80 (95% CI 0.57-1.09) to 2.91 (95% CI 2.4-3.5) per 1000 person-years (Figure 1). The incidence rates according to age follow a biphasic pattern: a first peak at the age of 15 to 24 year is followed by a sharp rise above the age of 45 (Figure 2).

### Outcome after syncope

**Figure 3:**
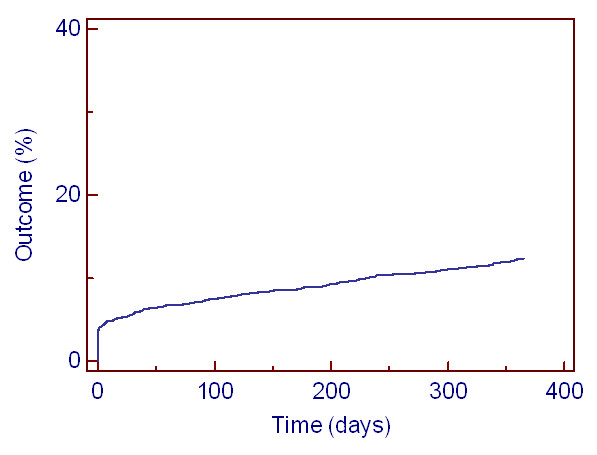
**Kaplan-Meier curve showing one year serious outcome after first syncope**.

As shown in the Kaplan-Meier curve, 12,3% of the patients with syncope have a serious outcome within one year (Figure 3). There was no difference in outcome when comparing male patients with female patients (HR 0.82 (95% CI 0.65-1.02). Patients having CV comorbidity and CV risk factors have a significant higher risk of serious outcome compared to patients without these CV risk factors (HR respectively 3.48 (95% CI 2.48-4.90) and 1.65 (95% CI 1.24-2.18)). Age is associated with serious outcome: a one-year increase in age is associated with 4% increase in hazard rate (HR 1.04 (1.03-1.04)). An age above 70 years or the presence of CV comorbidity increases the likelihood of serious outcome (positive likelihood ratio 2.22 (95% CI 2.05-2.41)).

### Comparing patients with and without syncope

Table 1 shows the characteristics of syncope and control patients. Syncope patients were more likely to have CV comorbidity (12.3% vs. 5.7%, P < 0.0001) and risk factors (18.7% vs. 11.3%, P < 0.0001). Mean age and gender were comparable in both groups.

**Table 1:** Characteristics of index and control patients

	Syncope N = 2785	Control N = 13909	
Mean age	54.5	54.5	NS
Male gender (%)	37.2	37.2	NS
CV comorbidity (%)	12.3	5.70	p < 0.0001
CV risk factors (%)	18.7	11.3	P < 0.0001

CV = cardiovascular

The Kaplan-Meier curve comparing syncope patients with control patients shows that syncope is a risk factor for serious outcome (HR 4.15 (95% CI 3.41-5.04)), with exception for the younger age group (< 23 years old) (HR 1.75 (95% CI 0.68 to 4.54), p = 0,17).

Cox regression, adjusting for age, gender, comorbidity and CV risk factors, showed that syncope on itself was a risk factor for serious outcome (HR 3.99 (95% CI 3.44-4.63)). The other independent risk factors were CV comorbidity (HR 1.81 (95% CI 1.51-2.17)) and age (HR 1.03 (95% CI 1.03-1.04)). Interaction terms comorbidity*syncope, age*syncope and age*comorbidity were non significant.

**Figure 4:**
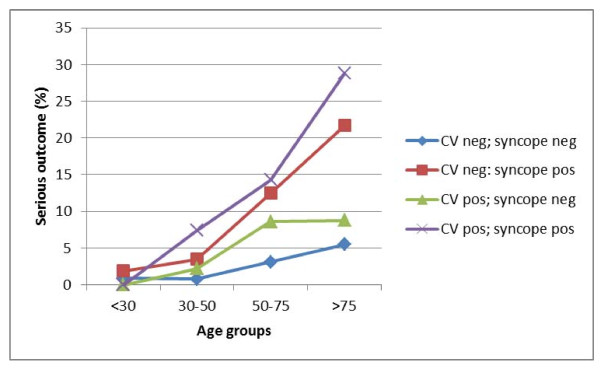
**One year serious outcome in first syncope and control patients according to CV comorbidity and risk factors**. 'CV pos': CV comorbidity OR risk factors prior to the baseline date.

Figure 4 shows the one year serious outcome in syncope and control patients according to CV comorbidity and CV risk factors in different age groups.

## Discussion

### Incidences

Our study results show an incidence of first syncope visits of 1.91 (95% CI 1.83-1.98) per 1000 person-years, increasing with age and with a clear gender difference (females 2.42 vs. males 1.40 per 1000 person-years). We choose deliberately to investigate only first syncope since patients with recurrent syncope are known to be at decreased risk for serious outcome [23]. Our overall incidence of syncope was 2.21 per 1000 person-years (95% CI 2.14-2.29).

Dutch general practice based data from LINH (Netherlands Information Network of General Practice) and the Amsterdam Transition Project show similar age and gender differences, but higher overall incidences (respectively 3.8 and 9.3 per 1000 person-years) [24,25]. A large population based study (mean age 51 (range 20-96) found an incidence of first syncope of 6.2 per 1000 person-years, age dependent with a sharp rise at 70 years, but with similar rates among men and women [8]. Keeping in mind that only 37% [5] to 56% [8] of patients with syncope seek medical attention (seeing a doctor or visiting a hospital) after syncope, our rates are comparable.

### Outcome after syncope

In our study, serious outcome within one year after syncope occurs in 12.3% of the patients.

In a general population-based study, outcome after syncope was dependent on the cause of syncope. One and 5 year mortality after cardiac syncope (including ischemia or arrhythmias) was approximately 15 and 45% respectively [8]. Previous prognostic studies of syncope in the ED show one year mortality ranging from 6% to 15.4% [12-14,26]. The STePS study, reporting on short and long term prognosis of syncope patients presenting in the ED, showed one year overall mortality of 6% and serious outcomes other than death (comprising cardiopulmonary resuscitation, pacemaker or ICD implantation, intensive care unit admittance and acute antiarrhythmic therapy) in 3.3% [12]. However, data collected in the ED syncope population cannot simply be extrapolated to general practice as was shown by Olde Nordkamp [9]. Using ED data in comparison to general practice data the author showed that the event rate for syncope in general practice (9.3/1000 patient-years) exceeded the presentation rate in the ED/chest pain unit by a factor 13.3 [9].

### Predictors for serious outcome

Predictors for serious outcome after syncope in our study are increasing age, CV comorbidity and CV risk factors. Our predictors of serious outcome after syncope are in keeping with literature data, originating from the ED. Abnormal ECG, history of ventricular arrhythmia, history of cardiovascular disease (including ischemic heart disease and congestive heart failure and TIA or stroke) were found to be independent predictors for serious outcome (including death and arrhythmias) for patients presenting with syncope in the ED [12,26,27]. Older age is associated with poor outcome, but the upper limit of low risk varies, from an age group > 45 years to the age of > 65 years (more often) [12,26,27]. Moreover, both coexistence of neoplasm and non-white race were found to be predictors for serious outcome [12,26]. Compared to a control group, syncope is a risk factor for serious outcome in our study, except in the younger age group (< 23 years old). This can result from insufficient power as both the incidence of syncope and the incidence of additional risk factors are lower in this age group. It can, however, also indicate a real absence of such an association. Although we are not aware of the causes of syncope in our patient population, reflex syncope is much more frequent than all other causes of syncope in the young and no increased mortality has been found in subjects that had suffered reflex syncope [28].

### Limitations

The Intego database does not provide mortality data nor information on signs, symptoms or additional test results e.g. ECG. Therefore, we were not able to test such information as either a measure of outcome or a possible risk factor.

The organization of primary care in Belgium is characterized by private system of health care delivery, based on independent medical practice, free choice of service provider and predominantly fee-for-service payment [29]. As several different categories of primary care organizations exist in and outside Europe [30], our data cannot be simply extrapolated to other countries.

### Implications

Although incidences of syncope in primary care are low, serious outcome after syncope is frequent. The knowledge of risk factors for serious outcome after syncope, based on all available evidence, seems to concentrate on age, CV risk factors and comorbidity. It is important that these risk factors seem largely similar over different settings (general population, general practice and ED). However current risk stratifying tools, developed in the ED, lack accuracy to determine patients at high risk for serious outcome. Further research should attempt to address the following question. How to detect patients with syncope that need immediate referral and additional testing. Does referral or hospitalization affect short- or long-term outcome?

## Conclusions

Incidence rate of first syncope in primary care was 1.91 per 1000 person-years. One year risk of serious outcome after syncope was 12.3%. Increasing age, CV comorbidity and risk factors are associated with serious outcome. Compared to a control group, syncope on itself is an independent risk factor for serious outcome (adjusted for age, gender, CV comorbidity and risk factors).

## Competing interests

The authors declare that they have no competing interests.

## Authors' contributions

PV carried out the analysis and drafted the manuscript. JBG, FB and BA participated in the study design and helped to draft the manuscript. SB provided the data and commented on the manuscript. All authors participated in revising it and approved the final version.

## Ethical approval

The Intego procedures have been approved by the ethical review board of the Medical School of the Catholic University of Leuven under N° ML1723.

## Pre-publication history

The pre-publication history for this paper can be accessed here:

http://www.biomedcentral.com/1471-2296/12/102/prepub
